# Evolutionary insights into toxins diversity in Ceriantharia (Cnidaria; Anthozoa)

**DOI:** 10.1016/j.toxcx.2025.100227

**Published:** 2025-06-04

**Authors:** Celine S.S. Lopes, Rafael E. Iwama, Thainá Cortez, Sónia C.S. Andrade, Anna M.L. Klompen, Jorge A. Audino, Jason Macrander, Adam M. Reitzel, Renato M. Nagata, Emilio Lanna, Lucas D. Martinez, Barbara M. Chagas, Sérgio N. Stampar

**Affiliations:** a Laboratório de Evolução e Diversidade Aquática - LEDA Lab/FC, Departamento de Ciências Biológicas, Universidade Estadual Paulista (UNESP), Av. Eng. Luiz Edmundo Carrijo Coube, 14-01 - Vargem Limpa, Bauru, São Paulo, Brazil; b Instituto de Biociências, Departamento de Zoologia, Universidade Estadual Paulista (UNESP), Rua Prof. Dr. Antônio Celso Wagner Zanin, 250 - Distrito de Rubião Junior, Botucatu, São Paulo, Brazil; c Laboratório de Diversidade Genômica - LDG, Departamento de Genética e Biologia Evolutiva, Instituto de Biociências, Universidade de São Paulo (USP), Rua do Matão, 277 - Cidade Universitária, São Paulo, São Paulo, Brazil; d Department of Ecology and Evolutionary Biology, University of Kansas, 1200 Sunnyside Ave., Lawrence, KS, 66045, USA; e Stowers Institute for Medical Research, 1000 E 50th St, Kansas City, MO, 64110, USA; f Departamento de Zoologia, Instituto de Biociências, Universidade de São Paulo, Rua do Matão, 101, Trav. 14., São Paulo, 05508-090, SP, Brazil; g Department of Biology, Florida Southern College, 111, Lake Hollingsworth Drive Lakeland, FL, 33801, USA; h Department of Biological Sciences, University of North Carolina at Charlotte, 9201 University City Blvd., Charlotte, NC, 28262, USA; i Laboratório de Zooplâncton, Instituto de Oceanografia, Universidade do Rio Grande (FURG), Avenida Itália km 8, Rio Grande, Rio Grande do Sul, Brazil; j Laboratório de Biologia Evolutiva e do Desenvolvimento - LABED, Instituto de Biologia, Universidade Federal da Bahia (UFBA), Rua Barão de Jeremoabo, s/n - Salvador, Bahia, Brazil

**Keywords:** Tube-dwelling anemones, RNASeq, Toxin-like genes, Development stages, Evolution, Functional genomics

## Abstract

Ceriantharians synthesize and inoculate the toxins found in their stinging cells spread throughout the body. For most cnidarians the putative toxins profile can vary widely depending on the tissue function and the environmental conditions faced by these marine invertebrates. Extensive gene duplications events have impacted the diversity of the toxins system of cnidarians and could explain the rapid emergence of novel toxins. On the other hand, it seems for Ceriantharia, the putative toxins profile does not exhibit major variation, despite occupying different ecological niches. Some species of ceriantharians have a planktonic stage that is highly dispersive, while the benthic phase is characterized by semi-sessile polyp. However, the polyp builds a tube involving the entire column that can play an additional function by protecting against predators and competitors, which could decrease the need to synthesize a wide array of toxins. In the present study, we compare the putative toxins of the larva and polyp of *Arachnanthus errans* based on the functional annotations of the transcriptomes against annotated protein databases. We seek to understand the evolutionary process of two toxin-like protein families using phylogenetic reconstruction methods with target sequences of the transcriptome of nine ceriantharian species. Our exploration revealed that the larva expresses 70 more toxin-like genes than the polyp, which may relate to abiotic and biotic factors the larva experiences. Our phylogenetic analyses suggest duplication events may have occurred in both toxins-like proteins and the two copies of *Kunitz*-like proteins might have been present in the common ancestor of Ceriantharia.

## Highlights

• The larva and polyp of *A. errans* have different putative toxins profiles.
• Genes associated with response to environmental stress were found in *A. errans.*
• Genes related to metamorphosis were identified in the larva transcriptome.
• Putative functions of reproduction and immunity were found in the polyp's genes.
• ShK-like and Kunitz-type toxins in Ceriantharia underwent duplication events.

## Graphical abstract

**Figure:**
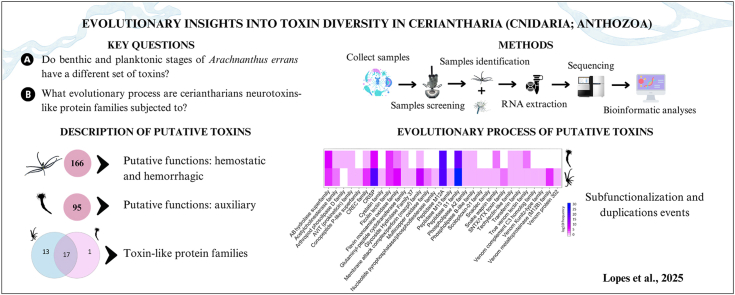

## 1 Introduction

The phylum Cnidaria represents the earliest diverging animal lineage of venomous taxa and serves as a suitable group to understand the adaptive dynamic and evolutionary origins of the toxin and toxin-like genes (Park et al., 2012; Jouiaei et al., 2015; Sunagar and Moran, 2015). The synthesis and inoculation of toxins by specialized sting cells, the cnidocytes (Kass-Simon and Scappaticci, 2002; Fautin, 2009) that are spread throughout all tissues, was an evolutionary novelty that contributed to the adaptive success of the cnidarians (Macrander et al., 2015; Huang et al., 2016; Jaimes-Becerra et al., 2019). As the production of toxins can occur across the entire body in all cnidarians, the putative toxins profile may vary in accordance to tissue function (Macrander et al., 2016; Ashwood et al., 2022; Klompen et al., 2022; Babonis et al., 2023). However, there are exceptions; some sea anemones (Actiniaria) produce toxins in both nematocysts and ectodermal gland cells (Moran et al., 2012). Unlike medusozoans, organisms of the subphylum Anthozoa spend most of their lives as semi-sessiles, making toxins used for defense and predation indispensable (Moran et al., 2009). While lacking a medusoid life stage, anthozoans can move widely along the coast during the larval phase (Ball et al., 2002; Zhou et al., 2017). For example, the larval stage of some species of the subclass Ceriantharia has the potential to achieve dispersal distances of more than 4000 km (Stampar et al., 2015a), which likely contributed to the emergence of morphological, physiological and behavioral traits to respond to drastic environmental changes and the challenge of dispersing long distances (Nyholm, 1943; Stampar et al., 2015a). Such capacity of dispersal can expose these larvae to variable environmental conditions, including temperature (9 − 29 °C), salinity (31 − 36 ppt), habitat availability (Tosetto et al., 2022; Lopes et al., 2023a), and predator exposure, which may request for a specific toxin arsenal. In contrast to the high mobility of the larva, the adult stage usually has low ability to move, living in a tube built from ptychocysts (Ceriantharia-specific cnidocyte sturctures) and marine sediments (Stampar et al., 2015b). While the polyp is, probably, exposed to less environmental variations, the low mobility may have made the ceriantharians more susceptible to predators and prevented the active search for food. The tubes of ceriantharians are hypothesized to be a functional structure used for protection (Stampar et al., 2015b), which may reduce predation. Despite mechanisms for tube formation being similar across Ceriantharia, the tube's thickness, length, and the overall architecture differ at the family level (Stampar et al., 2015b). Specimens of the families Arachnactidae and Botrucnidiferidae, usually build simpler thin and fragile tubes, while members of the family Cerianthidae make thick (up to 3 cm) intricate tubes, which can have multiple openings and lengths which can reach twice the length of the specimen's body (Frey, 1970; Stampar et al., 2015b). While the tube is an additional novel trait to give protection to tube anemones, it does not completely eliminate challenges these species face when considering predation and efficient prey capture. Probably these marine invertebrates had to develop additional strategies to deal with predators and to catch prey. One effective strategy for both prey-capture and predator deterrence is the production and deployment of different arsenals of toxins (Moran et al., 2012; Yosef et al., 2020; Klompen et al., 2020). However, the genomic repertoire underlying the emergence and evolution of toxins in Ceriantharia are still poorly understood.

The toxin-like genes profile for the polyp stage was evaluated for four species within Ceriantharia and showed no remarkable variation from toxins found in other cnidarians (Klompen et al., 2020). However, for most species of Ceriantharia, the toxin repertoire across diverse taxa and developmental stages remains unknown. The extreme contrast in established niche, behavior, and range of dispersion between ceriantharian's larva and polyp raise questions about variation in the putative toxins of these two development phases. Klompen et al. (2020) identified toxin families that are very commonly found in anthozoans, such as *ShK-domain* and *Kunitz-domain* protease inhibitors. These toxins play crucial roles in biological processes essential for survival of the organisms that synthetize them. The protease inhibitors are indispensable in the regulation of the protease activities and they participate in some signaling pathways (Rawlings et al., 2004). The *kunitz-domain*, which is abundant in venom of cnidarians (Liao et al., 2017; Klompen et al., 2020; Xiao et al., 2022), can act as protease inhibitor, ion channel blocker and other inhibitory functions (Mishra, 2020). The *Stichodactyla helianthus* K channel toxin (*ShK-like*) is a family within the superfamily *ShKT* that is involved in morphogenesis, regeneration and cell differentiation (Shafee et al., 2019). Although the sequences of *ShK-like* domains are divergent, their structures are very conserved (Shafee et al., 2017, Shafee et al., 2019).

Even though the toxin profiles have been increasingly investigated in cnidarians, the evolutionary processes responsible for its emergence and the changes over time needs further investigation. Toxins experience adaptive pressure and positive selection is hypothesized to strongly act in the genes-encoding toxins even in ancient lineages such as Cnidaria (Casewell et al., 2013; Gacesa et al., 2015; Klompen et al., 2021). However, Sunagar & Moran (2015) defended the divergence in the evolutionary route of venoms between the ancient and recent lineages; while the genes-like toxins of the youngest clades are submitted to positive selection, in the ancient clades these genes suffer influence of negative selection. Based on this scenario, mechanisms that contribute to a fast adaptation to novel environmental conditions would be important. Gene duplication can provide variation in toxin profiles through dosage or divergence of paralogous loci, changing the gene expression, which can result in variation in the phenotypic traits (Smith et al., 2023). This could be useful to toxin-like genes, since they need to respond quickly to shifts in niches.

In recent years, several studies have sought to understand how toxins evolved to explain processes shaping the diversity of venoms across diverse lineages (eg. Moran et al., 2008a; Casewell et al., 2011; Jouiaei et al., 2015; Surm et al., 2019). The “birth-and-death” model of gene family evolution has been used to explain the emergence of venoms (Nei et al., 1997; Fry et al., 2009; Sachkova et al., 2020). This hypothesis states that the emergence of toxin-like genes families can occur by convergent recruitment of genes involved in non-toxic functions, followed by duplication events and independent mutations at accelerated rates in different organisms groups, which could partially explain the diversity of toxins (Nei and Rooney, 2005; Fry et al., 2009; Casewell et al., 2011). On the other hand, in more specific cases, such as the multigene family encoding neurotoxins genes *Nv1* clusters in *Nematostella vectensis*, the low nucleotide diversity was explained by concerted evolution (Moran et al., 2008a). The concerted evolutionary model assumes the genes of a family do not evolve independently, instead they evolve in consonance; so that when a mutation occurs in one copy of the gene, it is spread to the other copies by gene conversion or unequal crossover (Brown and Sugimoto, 1974; Nei and Rooney, 2005). This model can explain how paralog genes in the same species are more similar than their respective orthologs in a distinct species (Nei and Rooney, 2005; Moran et al., 2008a). Later, concerted evolution was found acting in other neurotoxins genes in Actiniaria (Moran et al., 2009; Smith et al., 2023). Besides that, evolutionary processes like fusion and recruitment acting in the neurotoxins Type I and III in *Anemonia viridis* explain the similarity in the function of these two toxins (Moran et al., 2009).

To bridge the gap in understanding the evolution of toxins in ceriantharians, here we provided the first investigation of the transcriptome of two different development stages of their life cycle. Our analyses were conducted following two approaches. We first describe and compare the transcriptome of the ceriantharian *Arachnanthus errans* to explore the differences between planktonic larvae and benthic polyp stages from the functional genomic point of view. We hypothesize that there is a remarkable difference in the composition of genes-like toxins content in the larva and polyp of *A. errans* because of the distinct niches experimented by them. Further, we explored the profile of toxin-like genes in the polyp stages of nine species of Ceriantharia, seeking to understand the evolutionary processes acting in the toxin-like proteins *Kunitz-type* and *ShK-like* throughout the three families of Ceriantharia.

## 2 Material & methods

### 2.1 Samples and tissue collection

Larvae of *A. errans* were obtained through trawling plankton nets with mesh sizes of 200 and 300 μm and mouth openings of 30 and 60 cm, respectively. Trawls were performed between January and March 2019, within the surf zone of Cassino Beach, located in Rio Grande - RS, Brazil (32° 16'and 52° 18′). Both, larvae and polyps were identified throughout morphological studies conducted for the species' description (Lopes et al., 2023b). During samplings, environmental parameters such as salinity and temperature were measured. Subsequently, the larvae were kept in the laboratory in 1 L glass containers filled with ∼3 cm layer of sand and ∼10 cm of seawater under stable salinity (30–34 ppt) and temperature (22° – 23 °C), as observed in the field. The larvae were fed daily with newly hatched nauplii of *Artemia* sp. until they progressed to the mature polyp stage to be used in this study. Polyps of the following species were collected between the years 2017 and 2019 by scuba diving: *Ceriantheomorphe brasiliensis* (Cerianthidae), *Isarachnanthus nocturnus* (Arachnactidae) (São Sebastião, São Paulo State), *Pachycerianthus* cf. *maua* (obtained from Discovery Place Science, Charlotte, NC, USA), *Pachycerianthus borealis* (purchased from Gulf of Marine Inc. in Pembroke, ME, USA) have the sequences obtained from the study of Klompen et al. (2020), *Pachycerianthus magnus* (Buleleng, Bali, Indonesia), *Isarachnanthus maderensis* (Porto Moniz, Madeira Island, Portugal), *Botruanthus mexicanus* (Sisal, Mexico) and *Ceriantheopsis americana* (Tampa Bay, Tampa, USA). For this study only one specimen in the polyp stage of each species and one larva from *A. errans* were used for Illumina sequencing and posterior analyses. Tissue samples from the marginal tentacles were stored in the RNAlater Stabilization solution (Qiagen, CA) and maintained at −80 °C. The field collections carried out in Brazil were approved by Instituto Chico Mendes de Conservação da Biodiversidade – ICMBio and Sistema de Autorização e Informação em Biodiversidade – SISBIO (project number: 72673-1).

### 2.2 Construction of cDNA library and RNA sequencing

RNA was separately extracted from one larva of *A. errans* and nine polyps, one of each species, with the RNAqueous Total RNA Isolation Kit® (Thermo Fisher Scientific, Massachusetts, USA) following the manufacturer's protocol. RNA quality was assessed using NanoDrop™ 2000 Spectrophotometer (Thermo Fisher Scientific). The quantification of total RNA was performed with Quant-it™ RiboGreen RNA Assay Kit (Thermo Fisher) and the integrity was evaluated by Agilent Bioanalyzer 2100 system (Agilent Technologies, Santa Clara, CA, USA). The high-quality total RNA was used to prepare the library using the TruSeq® Stranded RNA Library Prep Kit (Illumina, San Diego, CA, USA) following the manufacturer's protocol and sequencing was performed on Illumina HiSeq 2500 system. The raw sequences of the larva and polyps were submitted to the Sequence Read Archive (SRA) on National Center for Biotechnology Information (NCBI) under BioProject accession number: PRJNA1227202 and PRJNA1030146.

### 2.3 De novo transcriptome assembly and assessment of quality

The approaches described above were conducted for all dataset studied here, one larva of *A. errans* and nine polyps, one of each different species. The quality of the raw sequencing reads was verified with FastQC v. 0.11.8 (Andrews, 2010). Low quality reads (cut-off <30 *phred* score) and adapter contamination were filtered using Trimmomatic v. 0.38 (Bolger et al., 2014). The rRNA was removed with the Silva database (Quast et al., 2013). The remaining reads were submitted to Trinity v2013-8-25 (Grabherr et al., 2013) for *de novo* assembly separately with default parameters, using reads originating from transcriptomes sequencing of one larva only of *A. errans* and of the nine polyps. The completeness and quality of the assembly was assessed with BUSCO v. 5.8.2 (Simão et al., 2015) based on the Metazoa database (N = 978 genes) and ExN50 statistics, respectively. The CD-HIT v. 4.8.1 was used for clustering of proteins with similarity higher than 98 % (Fu et al., 2012).

### 2.4 Functional annotation

For all transcriptomes analyzed here, candidate coding regions were identified with TransDecoder v.5.5.0 (Haas, 2018). Functional annotations of the transcriptomes were performed using InterProScan v. 5.72–103.0 (Jones et al., 2014). The assembled transcripts were annotated based on homology search against known databases (SwissProt, NCBI), protein domain identification, using HMMER (Finn et al., 2011) against the Pfam database (Finn et al., 2014). The signal peptide and transmembrane region prediction were verified using SignalP (Teufel et al., 2022) and TMHMM (Krogh et al., 2001). Additional searches against annotation databases, such as Kyoto Encyclopedia of Genes and Genomes - KEGG (Kanehisa et al., 2022) and eggNOG (Huerta-Cepas et al., 2019) with cut-off E-value 1e-5 were conducted. The transcripts were further annotated using Gene Ontology – GO terms to describe functions of the encoded transcripts at biological, cellular, and molecular levels.

### 2.5 Annotation of putative toxin-like gene

The annotation of toxin-like genes was conducted based on two different approaches: (a) the first one had the objective to identify and describe the set of putative toxins of the larva and polyp only of *A. errans*; (b) the second one was guided to select only target toxin-like genes (*Kunitz-type* and *ShK-like*) to conduct the phylogenetic reconstructions. The identification of the toxin-like genes to identify the putative toxin in the larva and one polyp of *A. errans* (first approach) was based on the modified protocol from Klompen et al. (2020). For each assembled transcriptome of one larva and all nine polyps of different species, protein coding regions were predicted via Transdecoder with a length cutoff of 50 bp. Only coding regions with start and stop codons were used in subsequent analyses. A multi-search strategy was conducted using BLASTp v.2.11.0 via BLAST+ (Altschul et al., 1997; Camacho et al., 2009) and *hmmsearch* via the HMMER v3.3.2 software suite (Finn et al., 2011; Potter et al., 2018). Briefly, BLAST databases were constructed for the transcriptome of the larva and the nine polyps. Searches using the BLAST database were performed against ToxProt (downloaded in March 2021 and July 2024) (Jungo et al., 2012). Subsequently to general search against the Tox-Prot, two other more specific databases were built from SwissProt/Uniprot with search filters by taxonomy and protein name. The filtering was performed using the following terms: (1) (Cnidaria AND (*ShK*)) and (2) (Cnidaria AND (*Kunitz*)) and the results were downloaded in July 2024. The last two databases were BLASTed, separately, against the nine assembled transcriptomes of the polyps to identify the target sequences of toxin-like proteins employed in the phylogenetic analyses. The cnidarian-specific NCBI dataset (Cnidaria AND (toxin) OR (venom)); downloaded in March 2021 was used to identify all toxins-like proteins of the larva and polyp of *A. errans*. All databases were built with an e-value cutoff of 0.001. Each assembly of the larva and polyp of *A. errans* was also searched with *hmmsearch* using custom Hidden Markov model libraries modified from Klompen et al. (2020) and von Reumont et al. (2014) (e-value cut-off = 0.001) and additional searches were performed against models for four families of venom cnidarian-specific from VenomZone (https://venomzone.expasy.org/; accessed March 2021): NaTypeI, NaTypeII, KType1a, KType1b, KTypeIII. For the first approach, the SignalP v5.0 server (Armenteros et al., 2019) was used to predict candidates with predicted signal peptides and filter the toxin candidates based on the datasets of the larva and polyp of *A. errans*. While for the second approach the SignalP was used only to predict signal peptides. *BLASTp* tool was used to perform a comprehensive search (e-value cutoff = 1e^5^) against Tox-Prot and Ref-Seq non-redundant proteins – NCBI (all downloaded April 2021 and July 2024) (Pruitt et al., 2005), and *hmmsearch* against Pfam (downloaded April 2021 and July 2024) (Sonnhammer et al., 1997) with the same e-value cutoff for all assembled transcriptome (larva and polyps), separately. The results were manually verified to validate matches between the Tox-Prot and SwissProt/Uniprot (filtered database by taxonomy and protein name) annotations and the associated domain identified from Pfam. Additionally, sequences that matched a *ShK domain* but did not match a ToxProt sequence were manually evaluated to ensure the predicted domain contained the characteristic six cysteine residues, and were included in downstream analysis of the larva and one polyp of *A. errans*.

Toxin candidates that did not match in the Tox-Prot searches, classified as uncharacterized/non-predicted protein, or lacking a toxin domain were discarded from additional analyses. The toxin candidates of the larva and polyp of *A. errans* were classified according to protein families, molecular functions and putative biological functions.

### 2.6 Alignments and phylogenetic analysis

For the *Kunitz-type* and *ShK-like* families, the alignments (Supplementary materials) were performed with the protein sequences of each putative toxin family for nine polyps species using the L-INS-I algorithm in MAFFT v. 7 (Katoh and Standley, 2013). The *Kunitz-type* and *ShK-like* trees were rooted with protein sequences of *Stichodactyla haddoni* (UniProt access number: B1B5I8) and *Nematostella vectensis* (UniProt access number: A7ST80), respectively. IQTREE v. 2.3.6 (Nguyen et al., 2015) was used to predict the best model-fitting using ModelFinder v. 1.5.4 (Kalyaanamoorthy et al., 2017) and to reconstruct gene trees under Maximum Likelihood (ML). The best fit model for *Kunitz-type* tree reconstruction was WAG + F + R3 and for *ShK-like* trees was WAG + G4. The default sets and 1,000 replicates to search for the best trees were used. The bootstrap supports values were calculated using 1,000 replicates.

## 3 Results

### 3.1 RNA sequencing and *de novo* transcriptome assembly

Three datasets were analyzed, containing i) larva x polyp transcriptomes of *A. errans*, ii) *ShK-like* genes from nine polyps of different species and iii) *Kunitz-type* protein from nine polyps of all species. Sequencing and assembly metrics of all ceriantharians transcriptomes used in this study are presented in Table 1. Briefly, the sequencing of the larva transcriptome resulted in ∼55,600,000 bp of raw reads. While the sequencing of the polyps transcriptome generated between 20,900,000 and 69,000,000 bp. After trimming based on quality and length, approximately 737,000 reads were removed from the larva sequencing; between 1,642,228 and 4,036,379 bp were excluded from the polyps sequencing. The assembled transcriptome of the larva was composed of 43,407 genes and 73,920 transcripts. For the polyps, our analyses recovered between ∼92,000–961,500 genes and ∼119,800 - 1,000,000 transcripts. The N50 statistics of the *de novo* assembled transcriptome of the larva was 1914 and BUSCO value was 95.8 %. The assembled transcriptomes of the polyps had a N50 between 380 and 1780 and BUSCO value of ∼3 %–43 %. The assembled transcripts of the larva and polyp of *A. errans* were BLASTed against four databases (Table S1). The searches with all polyps transcriptomes analyzed in this study against the database filtered by taxonomy (Cnidaria) and protein name (*Kunitz* or *ShK*) using *BLASTp* (Table S2) showed hits more significant than those searches against the ToxProt database filtered only by protein name (Table S3).

**Table 1:** Sequencing and assembly metrics of the nine polyps transcriptomes and one larva transcriptome of Ceriantharia. **bp** = base pairs. Raw reads from Klompen et al. (2020) are noted with ☨.

	Larva	Polyps								
	*A.errans*	*A.errans*	*I.nocturnus*	*I.maderensis*	*B.mexicanus*	*C.brasiliensis*	*C.americana*	*P.borealis*	*P.maua*	*P. magnus*
**Paired-raw reads (bp)**	55,699,224	42,781,312	31,028,274^☨^	61,536,383	69,185,842	34,877,883^☨^	20,935,195	36,520,791^☨^	27,865,720^☨^	25,875,655
**Reads after trimming (bp)**	54,961,433	40,214,006	29,023,716	58,281,881	65,446,394	32,831,924	16,898,816	34,650,424	26,223,492	22,033,305
**BUSCO score (%)**	95.8	73	80.4	91.7	68.2	90.6	3.1	98.8	76.5	11.4
**N50**	1914	1195	1247	1780	893	1279	380	1379	1100	1086
**GC content (%)**	44.03	43.04	43.01	43.06	37.92	37.43	35.63	38.51	37.20	36.02

### 3.2 In silico functional annotation of the larva and polyp stages of *A.errans*

**Fig. 1:**
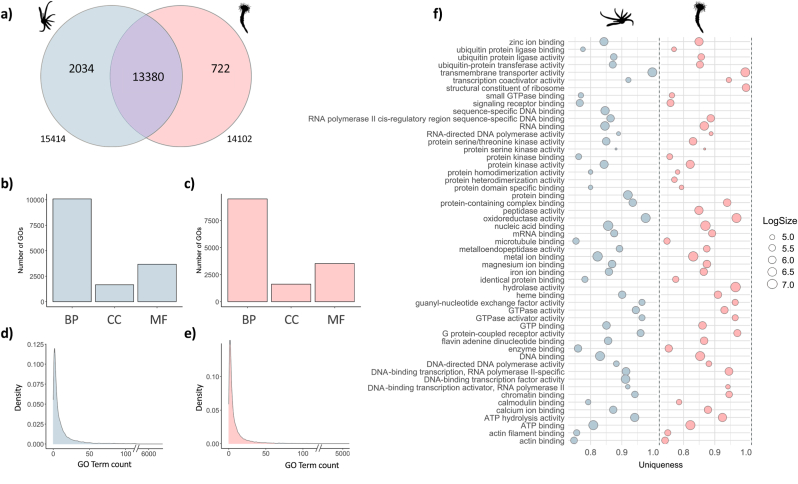
Gene Ontology (GO) classes matching with transcriptome of the larva and polyp of *A. errans*. **a.** Venn Diagram of the amount of terms GO associated with larva and polyp transcriptome. **b.** Number of GO terms identified to three GO categories in the larva transcriptome. **c.** Number of GO terms identified to three GO categories in the polyp transcriptome. **d.** Density of GO terms matching with genes of the larva transcriptome. **d.** Density of GO terms matching with annotated genes of the larva transcriptome. **f.** REVIGO analysis with the top 50 GO terms matching with larva and polyp transcriptome. **BP** = Biological process, **CC** = Cellular component, **MF** = Molecular function. Blue color corresponds to the larva. Pink color represents the polyp transcriptome. (For interpretation of the references to color in this figure legend, the reader is referred to the Web version of this article.)

**Fig. 2:**
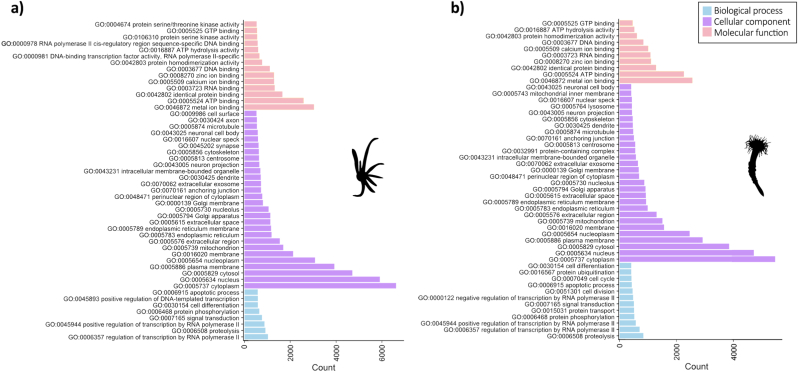
Top 50 of the GO terms matching with annotated genes of the larva and polyp of *A. errans*. **a.** GO terms corresponding to the larva transcriptome. **b.** GO terms corresponding to the polyp transcriptome.

Our analyses resulted in 11,644 and 19,731 unique genes annotated in the KEGG and Pfam databases, respectively, in the larva transcriptome. The annotations of the polyp transcriptome against KEGG and Pfam database generated 12,081 and 16,000 unique genes, respectively. In this study 15,414 and 14,633 unique categories of the GO terms were identified in the larva and polyp transcriptome, respectively (Table S1). The majority of GO terms, 13,380, were shared by both stages; 2,034 GO terms were attributed exclusively for the larva (L) and 722 were identified only for the polyp (P) stage (Fig. 1a). These GOs were hierarchically organized in “biological process”, “cellular component”, and “molecular function” (Fig. 1b–c). Our analysis was able to produce a total of 176,183 and 151,317 annotations for larva and polyp, respectively. The results generated 12,332 annotated unique genes for the larval stage and 11,138 for the polyp stage. Based on these annotated genes, most of unique categories were assigned to biological process in both life stages (L = 10,081; P = 9,493), followed by molecular function (L = 3,680; P = 3,524) and cellular component (L = 1,653; P = 3,524) (Fig. 1b–c). Furthermore, we selected the 50 most frequent GO terms associated with genes of the larva and polyp transcriptome annotated and analyzed them using REVIGO Web server (Supek et al., 2011). The profiles of GO terms more frequently annotated for the genes of both transcriptomes assembled were very similar (Fig. 1f). Between the GO terms with larger annotation number for both stages, we found “Transmembrane transporter activity”, “Oxidoreductase activity”, “Nucleic acid binding”, “Metal ion binding”, “DNA binding”, “ATP binding”, “ATP hydrolysis activity”, all in the molecular function category. However, our analyses evidenced three GO terms of the molecular function category associated only with genes of the larva: “Sequence-specific DNA binding”, “Protein binding” and “DNA-binding transcription factor activity”. Four GO terms of the molecular function category were found only correlated to genes of the polyp: “Structural constituent of ribosome”, “Protein heterodimerization activity”, “Peptidase activity” and “Hydrolase activity”. Approximately, half of the annotated genes were assigned to the GO term cytoplasm of the cellular component category in the transcriptome of both stages (Fig. 2a–b). We found most of the annotated genes associated with metal ion binding in the larva (∼3,500) and polyp (∼3,000) in the molecular function category. While, in the biological process, most genes were assigned to regulation of transcription by RNA polymerase II in the larva transcriptome (∼1,000) and proteolysis in the polyp stage (∼900).

### 3.3 Putative toxin-encoding transcripts of the larva and polyp of *A.errans*

**Fig. 3:**
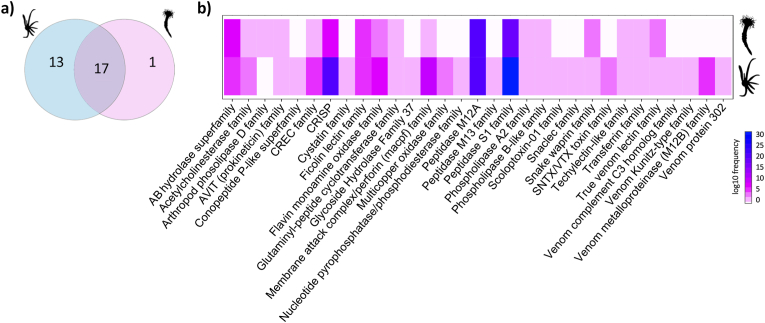
Families of toxin-like proteins of the larva and polyp of *A. errans*. **a.** Venn Diagram of the amount of families assigned to each development stage and quantity of families shared by larva and polyp phase. **b.** Families of putative toxins present and absent in both stages and frequency with which each family is attributed to transcripts at each stage of *A. errans*.

**Fig. 4:**
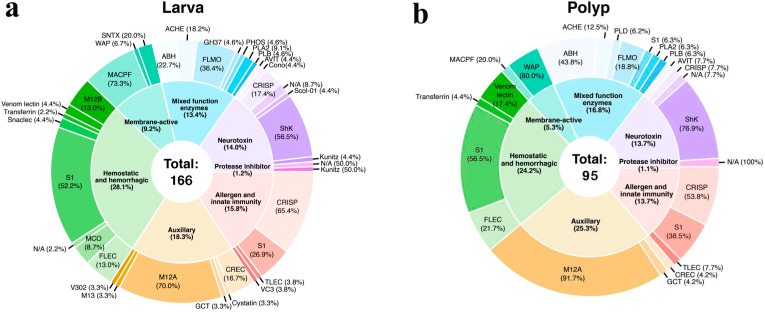
Toxin-like proteins matching with annotated genes and their functions for the larva and polyp transcriptomes. **Abbreviations:** ABH: AB hydrolase superfamily, ACHE: Acetylcholinesterase, AVIT: AVIT family, Cono: Conopeptide P-like superfamily, CREC: CREC family, CRISP: Cysteine-rich secretory proteins, Cystatin: Cystatin family, FLEC: Ficolin Lectin family, FLMO: Flavin monoamine oxidase family, GCT: Glutaminyl-peptide cyclotransferase family, GH37: Glycoside hydrolase family 37, Kunitz: Venom kunitz-type family, M12A: Peptidase M12A, MACPF: Membrane attack complex/perforin (macpf) family, M12B: Venom metalloproteinase (M12B) family, M13: Peptidase M13 family, MCO:Multicopper oxidase family, N/A: Non-identified protein family, PHOS: Nucleotide pyrophosphatase/phosphodiesterase family, PLA2: Phospholipase A2 family, PLB: Phospholipase B-like family, PLD: Arthropod phospholipase D family, S1: Peptidase S1 family, Scol-01: Scoloptoxin-01 family, Snaclec: Snaclec family, SNTX: SNTX/VTX toxin family, TLEC: Techylectin-like family, Transferrin: Transferrin family, V302: Venom protein 302, VC3: Venom complement C3 homolog family, Venom lectin: True venom lectin family, WAP: Snake waprin family.

A total of 30 and 18 different toxin protein families were identified in larva and polyp, respectively (Fig. 3a–b). The putative toxin-like proteins were clustered according to the functional annotation. Most of the annotated proteins were enzymes, structural proteins and toxin-like proteins (Table S4). Our analysis evidenced 13 exclusive putative toxin-like protein families in the transcriptome of the larval stage and only one protein family exclusively in the polyp phase (Fig. 3a). Our results found large gene copies encoding the same toxin-like protein families in the larval stage, for example, CREC family, CRISP and MACPF family, (Fig. 3b). For the same putative protein families in the polyp stage, the number of gene copies encoding them was smaller. In total, 260 coding transcripts likely associated with venomous elements were pinpointed across both stages. Specifically, the larval stage exhibited 166 expressed transcripts encoding toxins-like protein candidates, while the polyp stage only 95 (Fig. 4, Table S4). A higher proportion of transcripts with hemostatic and hemorrhagic function (n = 46) and auxiliary activities (n = 30) were identified in the larva of *A. errans* (Fig. 4a). While the inverse occurred in the polyp, putative toxins that play putative auxiliary role (n = 24) and hemostatic and hemorrhagic functions (n = 22) were more abundant (Fig. 4b)

### 3.4 Toxin-like phylogenetic relationships

The genes identified in the polyps of Ceriantharia as *ShK-like* toxins were similar to sequences from the Nematocyst *Expressed Protein 8* (NEP8) toxin family. However, we prefer to treat them more broadly as *ShK-like*. The alignment lengths of the sequences from *Kunitz-type* and *ShK-like* were 466 and 423 bp. The gene trees were reconstructed using sequences from the polyp stage that had hits against the database constructed only with cnidarian-specific and target toxins families sequences (Table S2). Our analyses from *ShK-like* toxins tree (-lnL 3854.275) recovered one clade constituted by two gene copies from species of different taxonomic families, Arachnactidae and Botrucnidiferidae (BS = 96) as a sister group of *B. mexicanus* (BS = 76). The clade of the genus *Pachycerianthus* (BS = 96) was a sister group to *I. maderensis* (BS = 46). The clade composed by *I. maderensis* + *P. borealis + P. maua* + *I. nocturnus* was found as a sister group of *A. errans* (BS = 84). Our results indicate duplication events might have occurred in *ShK-like* genes of the *I. nocturnus*.

**Fig. 5:**
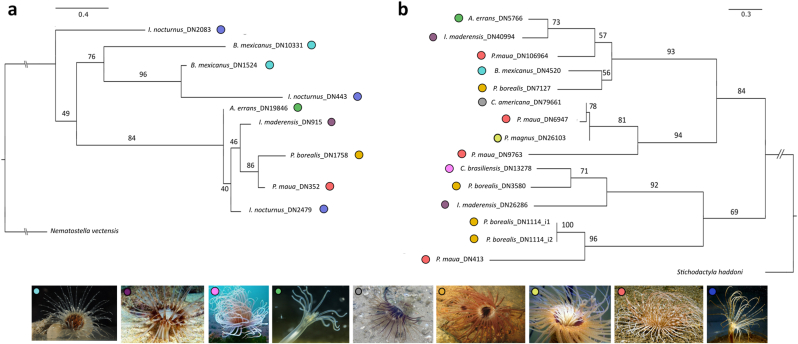
Maximum likelihood (ML) trees resulting from analyses of the ShK-like proteins and Kunitz-like proteins of the Ceriantharia. 1000 bootstrap replicates were performed for each analyses **a.** ShK-like tree (-lnL 3854.275). **b.** Kunitz-like ML tree (-lnL 3679.678). The colored circles represent each species and correspond to the colors disposed of in the figures below. (For interpretation of the references to color in this figure legend, the reader is referred to the Web version of this article.)

The gene tree resulting from the phylogenetic reconstruction with sequences corresponding to *Kunitz-type* protein (-lnL 3679.678) evidenced one clade with genes of two species of the family Arachnactidae (*A. errans* + *I. maderensis*) as sister group of a species of the family Cerianthidae (*P. maua*) (Fig. 5b, BS = 57). The clade composed by two species of the different families, Botrucnidiferidae (*B. mexicanus*) and Cerianthidae (*P. borealis*), was a sister group to the clade formed by *A. errans* + *I. maderensis* + *P. maua*, all comprising a clade with high bootstrap support value (BS = 93). One of the largest clades was identified only with species of the family Cerianthidae (*C. americana* + *P. maua* + *P. magnus* + *P. maua*) (BS = 94). One clade formed by *C. brasiliensis* + *P. borealis* (BS = 71), both from the same family, and *I. maderensis* was distinguished in our analyses (BS = 92) and was sister group of the clade composed by *P. borealis* and *P. maua* (BS = 69). Our phylogenetic analyses suggest the common ancestor of Ceriantharia likely had two copies of the Kunitz-type protein. We identified sequences of genes derived from the same taxa (*I. maderensis*, *P. maua* and *P. borealis*) appear in different clades, which can suggest gene duplication events occurring in *Kunitz-type* protein family in Ceriantharia.

## 4 Discussion

In this study, we describe the toxin-like proteins found in the transcriptome of the larva and polyp of *A. errans* through RNA-Seq analyses. The GO enrichment analysis more frequently attributed terms for the genes of both stages that were usually associated with the response to the environmental stress in other marine invertebrates. Although we have analyzed a low number of individuals in this study, the larva of *A. errans* was found in environments susceptible to rapid biotic and abiotic changes. We identified a higher amount of putative toxins in the larva (166) than polyp transcriptome (95), which can be related to different niches occupied by specimens from both developmental stages. We found some toxin-like protein families in *A. errans* that may be under subfunctionalization process. Additionally, phylogenetic analyses were conducted using *Kunitz-type* and *ShK-like* toxin genes from nine individuals of different species. Our analyses suggest duplication events occurring in both toxin-like protein families in Ceriantharia.

### 4.1 Comparative functional annotation between larva and polyp of *A.errans*

This is the first investigation that aims to elucidate the composition of toxins-like genes of larva and polyp in Ceriantharia, in addition to expanding our characterization of toxins genes found more broadly across this poorly studied subclass. In a previous investigation, we described the life cycle of *A. errans*, showing that this species undergoes a developmental sequence characterized by a brief cerinula larval stage (lasting approximately seven days in the planktonic environment), with an agile swimming and feeding behavior (Lopes et al., 2023b). Moreover, during the polyp stage, *A. errans* demonstrates the behavior of exiting its tube, actively exploring its surroundings, and likely constructing multiple tubes over its lifetime (Lopes et al., 2023b). In the present study, we conducted a transcriptome analysis of both the larval and polyp stages of *A. errans*, providing functional annotations of expressed genes for these distinct developmental phases. Although the present study was conducted without replicates due to the difficulty in obtaining more samples because of the complexity in collecting larvae and polyps of Ceriantharia already documented, our results provide new insights about the putative toxins-like genes arsenal in two different development stages in Ceriantharia and can be used as a base for future studies.

When comparing larval and adult GO analyses, we found that the majority of the annotated genes were categorized under the "cytoplasm" GO term in both the larva and polyp stages (Fig. 2). The prevalence of the term "cytoplasm" in the developmental stages of other marine invertebrates has been shown to be related to innate immunity mechanisms and metabolic activities (Saco et al., 2021; Liudkovska et al., 2022). The most prevalent terms matching with annotated genes based on REVIGO analysis, “Transmembrane transporter activity”, “Metal ion binding” and “Nucleic acid binding” were related to responses to the environmental stress, such as decrease of salinity and alkaline level in Mollusca and Crustacea respectively (Quin et al., 2021; Gong and Li, 2023). The locality where the larva of *A. errans* was collected is characterized by estuarine dynamics that undergo rapid changes in salinity (Teixeira-Amaral et al., 2021). Therefore, in the larval stage, *A. errans* faces drastic changes in salinity and to develop mechanisms that ensure the osmotic pressure could be extremely relevant to survival. Another prevailing term was “Oxidoreductase activity”; which has been associated with genes acting in stress responses in invertebrates, such as the responses of the Pacific oysters of the species *Crassostrea gigas* to the increase of the seawater temperature (Chi et al., 2023) and *Whitmania pigra*, a species of leech to the freshwater environmental experimentally contaminated with lead (Kun et al., 2022). “DNA-binding” and “ATP binding” are also the most prevalent terms related with our annotated genes and were associated with the resistance of the oyster *Pinctada fucata martensii* to the experimental exposure to the titanium dioxide nanoparticles (Li et al., 2024). Indeed, water temperature from Patos Lagoon, where the larva of *A. errans* was collected, is highly variable, between summer and winter, ranging between 10 and 31 °C (Teixeira-Amaral et al., 2021). Our analyses suggest that both, larva and polyp, have genes associated with the similar GO terms that indicate response mechanisms to drastic abiotic variations.

Our REVIGO analysis identified three GO terms linked to genes annotated exclusively in the larval transcriptome, including “DNA-binding transcription factor activity” and “Protein binding”, both associated with marine invertebrate metamorphosis (Chandramouli et al., 2013; Yang et al., 2023). “Protein binding” was notably linked to transcripts abundant during larval development and metamorphosis in *Pseudopolydora vexillosa*, a polychaete that secretes mucus for tube building (Chandramouli et al., 2013). While metamorphosis might not be the best term to describe the change from the larva to juvenile polyp in *A. errans*, as this species undergoes subtle morphological modifications between both phases (Lopes et al., 2023b), larval samples near settlement likely captured stage-specific gene expression. Conversely, four GO terms were prevalent in the polyp transcriptome, including “Peptidase activity” and “Hydrolase activity”, which are closely related, as peptidases cleave peptide bonds in proteins (Rojo-Arreola et al., 2020). These enzymes play roles in immunity, development, reproduction (Leyria et al., 2018), and toxin-like protein formation (Klompen et al., 2020; Rodrigo et al., 2021). Our GO-based annotation provides insight into the functional traits spanning *A. errans*’ life cycle, potentially linking them to adaptive evolution.

### 4.2 Profile of putative toxins in the different development stages of *A. errans*

The ecological niche of marine invertebrates can vary significantly across distinct developmental stages (Cowen and Sponaugle, 2009). The pelagic larvae are exposed to variable biotic and abiotic factors that can impact their ability to capture prey, avoid predation, disperse, and other essential behavioral traits (Bosch et al., 2014; Griffith et al., 2021). Conversely, benthic organisms often employ diverse ontogenetic strategies over evolutionary time frames influenced by different environmental conditions (Love, 2010; Minelli and Fusco, 2010). Shifts in the cnidarian toxin-like gene expression profiles in each developmental stage may be a response to the distinct environments experienced during a specific ontogenetic stage (Ames et al., 2016; Columbus-Shenkar et al., 2018). *Arachnanthus errans* larvae have exclusively been discovered in estuarine coastal waters varying from 33 to nearly 0 ppt within a matter of hours due to continental freshwater inputs (Teixeira-Amaral et al., 2021). These events, combined with coastal currents, have the potential to transform the planktonic community, transitioning from a coastal-oceanic composition to a limnic one (Teixeira-Amaral et al., 2017). Additionally, the customary coastal currents, averaging approximately 0.5 m per second (Jung and Toldo Jr, 2011), have the potential to transport these larvae hundreds of kilometers away from their original beach habitat within a few days or week to adapt to such these environmental fluctuations, *A. errans* larvae may necessitate a diverse array of biochemical mechanisms after becoming subjected to different environmental conditions. At the polyp stage *A. errans* often employs locomotion and has built its tube (Lopes et al., 2023b), where it can hide when it is not in locomotion. Although the tube is thin, we hypothesized it as an additional protection to the polyp, mainly when analyzed together with the rapid locomotion of the polyp.

Our examination revealed distinctions in the toxin-like gene descriptions between the larval and polyp stages. The contrasting presence/absence of toxin-like genes across the larval and polyp stages of *A. errans* through our comparative transcriptome analysis identified 71 more toxin-like transcripts associated with the larvae stage when compared to the polyp stage (Fig. 4). The dynamic venom composition throughout the life cycle is recorded in Anthozoa (Moran et al., 2008b; Columbus-Shenkar et al., 2018), which is explained by the lack of nematocyst charged of neurotoxins during embryo and planulae stages and the adhesive rather than predatory function of nematocysts at these stages (Kass-Simon and Scappaticci, 2002). Although the initial larvae of *A. errans* do not feed when close to settlement, they have the ability to catch prey (Lopes et al., 2023b). We recovered 13 toxins-like protein families from the larval stage, a majority with putative hemorrhagic and hemostatic functions, including metalloproteinase 12B (M12B), snaclec, and multicopper oxidase families. Putative toxins of the M12B family were previously reported in Anthozoa (Gacesa et al., 2015), Scyphozoa (Liu et al., 2015) and Cubozoa (Hwang et al., 2022). M12B is the most abundant compound in the venom of the two scyphozoans, *Nemopilema nomurai* and *Cyanea capillata*; metalloproteinase-containing venoms are responsible for the wheal and blistering emergence (Wang et al., 2019). In Anthozoa, this family is probably associated with toxin maturation (Gacesa et al., 2015). Venoms from the snaclec protein family are commonly found in snakes interacting with coagulation factors (Clemetson, 2010), but also in Zoantharia, playing a role in hemostasis activities (Huang et al., 2016). Jaimes-Becerra et al. (2017) reported that snaclec is a compound abundant in the toxin profiles of the Scyphozoa, Anthozoa and Hydrozoa. Multicopper oxidase have been identified in the polyps of anthozoans, *N. vectensis* and *Acropora digitifera*, and in the hydrozoan *Hydra magnipapillata* with signaling to oxidative damage and metal toxicity responses (Shinzato et al., 2012). Our analyzes recovered multicopper oxidase family only in putative toxin description of the larva of *A. errans*. An arthropod phospholipase D protein was the only toxin-like protein family found exclusively by polyp of *A. errans* (Fig. 3). Although phospholipase roles in cnidarian venoms remains poorly characterized, they have been identified broadly across the phylum (Nevalainen et al., 2004; Jaimes-Becerra et al., 2017), they have been associated with inflammatory process, digestion of phospholipids, and protection against pathogens (Nevalainen et al., 2008; Murakami et al., 2016).

Both larval and polyp stages share 17 putative toxin-like protein families (Fig. 3a), the most numerous being peptidases of the S1 family, metalloendopeptidases and CRISP. The S1 family is widely documented as a type of enzyme that plays several roles, including venom dissemination, and induction of the hemorrhagic process in the prey (Mukherjee, 2014). The peptidase S1 is reported for Scyphozoa, Anthozoa, and Hydrozoa, but is absent in Cubozoa (Jaimes-Becerra et al., 2017). Recently, peptidase S1 was identified in the toxins profile of species of the family Cerianthidae and Arachnactidae in Ceriantharia (Klompen et al., 2020). Probably, the S1 family proteins were a trait present in the common ancestor of Medusozoa + Anthozoa, which was subsequently lost in Cubozoa. The metalloendopeptidases are highly expressed in acrorhagi of non-aggressive and aggressive polyps of the sea anemone *Anthopleura elegantissima* (Macrander et al., 2015). In the hydrozoan *Hydractinia symbiolongicarpus*, these toxins are related to the immunity functions. The class Myxozoa also expressed transcripts encoding potential toxins of the metalloendopeptidase family, even with parasitic behavior (Americus et al., 2021). Transcripts coding toxins of the CRISP family were the second most abundant in the larva and polyp of *A.* errans according to the results of this study. The CRISP family is an essential component in snake venom, highly myotoxic, and regulates the inflammation processes (Tadokoro et al., 2020). Toxins identified as the CRISP family were previously reported from most classes of cnidarians (Macrander et al., 2015; Jaimes-Becerra et al., 2017; Klompen et al., 2020; Xiao et al., 2022).

In general, the profile of putative toxins exhibited by the larva and polyp of *A. errans* converged with the scenario previously proposed for cnidarian venoms. However, our analyses revealed some ambiguities. The transcripts of the *A. errans* larval form encoded the complement C3 homolog family, which was not found in the ceriantharian's polyps from other species in a previous study (Klompen et al., 2020). The ficolin lectin family, restricted to Medusozoa (Jaimes-Becerra et al., 2017), was coded by transcripts of the larva and polyp of the ceriantharian analyzed in this study. Several toxins usually identified in Anthozoa were not found in our analyzes for the larva and/or polyp of the tube-dwelling anemone *A. errans*, such as potassium and sodium channels toxins, and actinoporins. However, future studies with deeper RNA-sequencing and replicates for members of both developmental stages are necessary to confirm this pattern. The neurotoxins, one of the groups of toxins remarkable in sea anemones of the order Actiniaria (Macrander et al., 2015) were poorly represented from our analyzes of the transcriptome of the larva and polyp of *A. errans*, corroborating with the results found by Klompen et al. (2020) in the polyp stages of four species of ceriantharians.

### 4.3 Toxin repertoire across polyps of Ceriantharia

A total of 17 toxin-like protein families were identified in all ceriantharians (ceriantharians analyzed in Klompen et al., 2020 plus and *A. errans*), which can highlight the existence of some similar ecological functions throughout the different tube anemone taxa, despite variation in geographic distribution, behaviors, and developmental mechanisms specific for each species (Stampar et al., 2015a; Lopes et al., 2019; Stampar et al., 2020; Ceriello and Stampar, 2023; Santos et al., 2024). Most of the transcripts encoding toxin-like proteins identified in polyps of *I. nocturnus*, *P.* cf. *maua*, *P. borealis* and *C. brasiliensis* are putatively involved in hemostatic and hemorrhagic functions (Klompen et al., 2020), unlike toxins identified in the polyp of *A. errans*. Three toxin-like protein families were present in the transcriptomes of *P.* cf. *maua*, *C. brasiliensis, P. borealis* (species from family Cerianthidae) and *A. errans* (species from family Arachnactidae), but they were not found for *I. nocturnus* (species from family Arachnactidae) transcriptome. Transcripts of *I. nocturnus* encoded 30 toxin-like protein families (Klompen et al., 2020), 20 of these families were also present in the profile of putative toxins of *A. errans* polyp. Gilatoxin (trypsin) was one toxin-like protein family exclusively found in the transcriptomes of the species of the family Arachnactidae, *I. nocturnus* and *A. errans*.

The polyp of *A. errans* exhibited a higher number (95) of putative toxins than *I. nocturnus* (69), both from the same taxonomic family, Arachnactidae (Klompen et al., 2020). Based on our dataset, six toxin-like protein families were identified in the polyp of *A. errans* and were not recorded for the other ceriantharians used in the Klompen et al. (2020) study. If confirmed in further studies with more samples, this scenario could reflect a response to biotic and abiotic changes faced by *A. errans* due to the locomotor behavior of the polyp (Lopes et al., 2023b), such as exposure to predators and prey variation, which other ceriantharians in the same developmental stage may not be susceptible, as they usually do not move outside their tube. The utility of toxins in many venomous organisms may be a direct result from ecological processes or changes throughout their lives (Casewell et al., 2013), intraspecific and interspecific variation of venom composition has been attributed to a variety of factors, including different habitats, ontogenetic features, diets, and predators (Cipriani et al., 2017; Sousa et al., 2017; Casewell et al., 2020). Furthermore, in cnidarians, variations in the toxin profiles are recorded occurring even in different regions of the body in the same organism (e.g. Surm et al., 2019; Ashwood et al., 2021). However, other studies suggest low variation in the putative toxins profile between Ceriantharia and other cnidarians (Klompen et al., 2020). Therefore, more data and further analysis is needed into Ceriantharia toxin assemblages from a functional standpoint as it is possible key venom components that have evolved within Ceriantharia have simply not been functionally characterized yet.

### 4.4 Evolution of ShK-like and Kunitz toxins in Ceriantharia

Cnidarians use venom for predation, defense and habitat competition (Purcell, 1985; Arai, 2005; Macrander et al., 2015), including the ceriantharian tube anemones (Santos et al., 2024). Therefore, from an adaptive point of view, it is interesting that some toxins can rapidly diversify. Indeed, the dynamism and consequently the structural and biochemical changes of venom are correlated with environmental shifts, which is essential to ecological function of toxins (Surm and Moran, 2021; Smith et al., 2023). The most recent study supports that species of Ceriantharia have a non-specific diet, feeding on fish larvae and many invertebrates, not significantly changing with habitat (Santos et al., 2024). On the other hand, cnidarians also need to deal with a significant diversity of predators, many which have developed adaptive traits in response to the defense mechanism of cnidarians (Goodheart and Bely, 2016; Arai, 2005). Based on this scenario of constant predator/prey interactions, selective pressures can vary significantly across toxin genes, resulting in high genetic diversification or purifying selection. Adding complexity, the diversification of most toxin protein families are believed to evolve under a birth-and-death model (Nei et al., 1997; Sachkova et al., 2020). In some cases, species of different lineages of Cnidaria appear to be evolving under negative selection (Gacesa et al., 2015; Rachamim et al., 2015). Jouiaei et al. (2015) hypothesized the negative selection drives the evolution of the cnidarian toxins, given that the toxin sequences analyzed had high conservation rate and dN/dS values < 1.

Based on the phylogenetic reconstructions, we identified that toxin genes *ShK-like* and *Kunitz-type* in polyps of Ceriantharia might have experienced duplication events, which is extensively identified as being the event behind the emergence of toxin genes (Gacesa et al., 2015; Xiao et al., 2022; Smith et al., 2023). Besides gene family expansion (Zhang, 2003), gene duplication can mold the phenotype of venoms in actiniarian sea anemones at the micro and macroevolution level (Smith et al., 2023). Duplication events can be strictly related to quick adaptation to prey resistance (Lynch and Conery, 2000; Casewell et al., 2013) and can result in (a) neofunctionalization, the emergence of a novel function after duplication (Jackson and Koludarov, 2020); (b) pseudogenization or the loss of one copy; purifying selection will act only in one copy, the others are free to accumulate deleterious mutations (Zhang, 2003; Lallemand et al., 2020) and (c) sub-functionalization; multiple functions of the ancestor gene will be distributed across duplicated genes, changes in the regulatory sequences drive changes in the expression pattern of the copies, multiple copies are, therefore, maintained (Zhang, 2003; Birchler and Yang, 2022). Gene duplications events can generate evolutionary novelties relevant to adaptation of organisms (Babonis et al., 2022). Studies conducted with cone snails suggest the gene duplication precede the whole genome duplication (Pardos-Blas et al., 2021; Farhat et al., 2023). However, as we use transcriptome data, our results did not reflect this scenario. For seven families of toxin-like proteins, such as CREC, CRISP and MACPF families, we identified a distinct number of gene copies in the different development stages in *A. errans* (Fig. 3b), which can be evidence of subfunctionalization. Even though there is evidence of duplication in our findings, additional tests should be further conducted to confirm this hypothesis. Moreover, processes involved in the maintenance and diversification of these copies are yet to be understood.

## 5 Conclusions

In this study, we provide functional annotation of the transcriptome and the identification of putative toxin-like genes of *A. errans*, a species of the subclass Ceriantharia, known to exhibit a short-time cerinula and a polyp with remarkable locomotion. This is the first record of the larval stage transcriptome annotation in Ceriantharia. The most GO terms were found in transcriptomes of both the larva and polyp, where terms related to stress responses were most frequently found. This scenario is consistent with the environmental conditions that vary depending on where the larva was collected. On the other hand, we identified GO terms related to the development process assigned only to genes of the larva transcriptome. The terms only attributed to polyp's transcriptome were associated with reproduction.

Our study is the first discussing putative toxin-like genes in different development stages in Ceriantharia. Our results identified more toxin-related genes in the larva than polyp of *A. errans* (L = 165, P = 95). We hypothesized that despite the larva spending a short-time on the plankton environment, it is exposed to greater variations than polyp, because it is found in the dynamic estuarine environment and due to higher dispersal capacity. Our phylogenetic analyses suggest that duplication events occurred in the putative toxins *ShK-like* and *Kunitz-type* in Ceriantharia. Moreover, probably the common ancestor of Ceriantharia had two copies of toxins *Kunitz-type*.

Our initial findings raise some questions that still remain unclear, for example: What evolutionary processes are acting on the diversification and emergence of toxins in Ceriantharia? Do different ontogenetic processes influence the toxin-like genes profile of other species in Ceriantharia? Are there intraspecific differences in the toxin-like gene profile in Ceriantharia? Are there differences in the putative toxin-like proteins profile in larvae of the same species of Ceriantharia that spend distinct time on the pelagic environment? Does the composition of the cnida and amount of cnidae types influence the toxins-like protein profiles? Nonetheless, here, we presented an important initial step to deeply explore RNA-Seq data of different developmental stages in Ceriantharia and the evolutionary process leading to the emergence of novel toxins.

## CRediT authorship contribution statement

**Celine S.S. Lopes:** Writing – review & editing, Writing – original draft, Visualization, Validation, Software, Project administration, Methodology, Investigation, Formal analysis, Data curation, Conceptualization. **Rafael E. Iwama:** Writing – review & editing, Validation, Software, Methodology, Formal analysis, Data curation, Conceptualization. **Thainá Cortez:** Writing – review & editing, Visualization, Validation, Software, Formal analysis. **Sónia C.S. Andrade:** Writing – review & editing, Supervision, Resources, Methodology, Funding acquisition, Data curation, Conceptualization. **Anna M.L. Klompen:** Writing – review & editing, Validation, Software, Formal analysis, Data curation. **Jorge A. Audino:** Writing – review & editing, Validation, Software, Formal analysis. **Jason Macrander:** Writing – review & editing, Validation, Software, Resources, Funding acquisition, Formal analysis. **Adam M. Reitzel:** Writing – review & editing, Resources, Funding acquisition. **Renato M. Nagata:** Writing – review & editing, Resources, Funding acquisition. **Emilio Lanna:** Writing – review & editing, Data curation. **Lucas D. Martinez:** Writing – review & editing, Validation, Software, Formal analysis. **Barbara M. Chagas:** Writing – review & editing. **Sérgio N. Stampar:** Writing – review & editing, Supervision, Resources, Project administration, Methodology, Funding acquisition, Conceptualization.

## Ethics in publishing statement

I testify on behalf of all co-authors that our article submitted followed ethical principles in publishing.

All authors agree that:

This research presents an accurate account of the work performed, all data presented are accurate and methodologies detailed enough to permit others to replicate the work.

This manuscript represents entirely original works and or if work and/or words of others have been used, that this has been appropriately cited or quoted and permission has been obtained where necessary.

This material has not been published in whole or in part elsewhere.

The manuscript is not currently being considered for publication in another journal.

That generative AI and AI-assisted technologies have not been utilized in the writing process or if used, disclosed in the manuscript the use of AI and AI-assisted technologies and a statement will appear in the published work.

That generative AI and AI-assisted technologies have not been used to create or alter images unless specifically used as part of the research design where such use must be described in a reproducible manner in the methods section.

All authors have been personally and actively involved in substantive work leading to the manuscript and will hold themselves jointly and individually responsible for its content.

No approval of research ethics committees was required to accomplish the goals of this study because experimental work was conducted with an unregulated invertebrate marine species.

## Funding sources

This work was supported by the Coordenação de Aperfeiçoamento de Pessoal de Nível Superior – Brasil (10.13039/501100002322CAPES), the National Council of Scientific and Technological Development (10.13039/501100003593CNPq -Research Productivity Scholarship) [grant number 301293/2019-8 and 304267/2022-8], the São Paulo Research Foundation (10.13039/501100001807FAPESP) [grant number 2017/50028-0; 2019/03552-0; 2022/16193-1; 2021/06738-8], and the Bahia Research Foundation (10.13039/501100006181FAPESB) [grant number INC0006/2019]. This is a contribution of the Brazilian Long-Term Ecological Research Program (BR-LTER), through the grants (10.13039/501100003593CNPq 441492/2016-9 and 442206/2020-8), State of Rio Grande do Sul Research Support Foundation (10.13039/501100004263FAPERGS 2551-0000102-2 and 2551-0000774-5) and 10.13039/501100014213INCT-Mar COI, funded by 10.13039/501100003593CNPq (610012/2011-8). The funding sources have no role related to study design, collection, analysis and interpretation of data, writing of the report and decision to submit the article for publication.

## Declaration of competing interest

The authors declare that they have no known competing financial interests or personal relationships that could have appeared to influence the work reported in this paper.

## Data Availability

All data generated and analyzed during this study are included in this published article and its supplementary information files. Raw sequences generated in this study are available at National Center for Biotechnology Information – NCBI under registration number: PRJNA1227202 and PRJNA1030146. Additionally, whenever requested, the data will be made available.
