# Supplementary material for: Evolutionary insights into toxins diversity in Ceriantharia (Cnidaria; Anthozoa)
Source: Toxicon X. 2025 Jun 4;27:100227. doi: 10.1016/j.toxcx.2025.100227 (PMC12182779; doi:10.1016/j.toxcx.2025.100227)
Supplement: Multimedia component 5 [file mmc5.docx]

**Supplementary material captions**

**Table S1.** Genes annotation of the larva and polyp transcriptomes of *A. errans*.

**Table S2.** *Blastp* against ToxProt database filtered by taxonomy (Cnidaria) and by protein name (*Kunitz-type* and *ShK-like*) for nine species of Ceriantharia.

**Table S3.** *Blastp* against ToxProt database filtered by protein name (*Kunitz-type* and *ShK-like*) for nine species of Ceriantharia.

**Table S4.** Custom annotation of toxin-like proteins for the larva and polyp of *A. errans*.
